# From Leaky Gut to a Vulnerable Brain: Obesity-Associated Gut Barrier Failure in Colorectal Cancer and Cognitive Dysfunction

**DOI:** 10.3390/nu18121909

**Published:** 2026-06-12

**Authors:** Soo Young Lee, Sang Hee Cho, Juhyun Song

**Affiliations:** 1 Department of Surgery, Chonnam National University Hwasun Hospital, Chonnam National University Medical School, Hwasun 58128, Jeollanam-do, Republic of Korea; syleecrs@jnu.ac.kr; 2 Division of Hemato-Oncology, Department of Internal Medicine, Chonnam National University Hwasun Hospital and Chonnam National University, Gwangju 61469, Republic of Korea; shcho@jnu.ac.kr; 3 Department of Anatomy, Chonnam National University Medical School, Hwasun 58128, Jeollanam-do, Republic of Korea

**Keywords:** obesity, colorectal cancer (CRC), gut–brain axis, intestinal barrier dysfunction, cancer-related cognitive impairment (CRCI)

## Abstract

Obesity is a major risk factor for colorectal cancer (CRC) and is increasingly recognized as a contributor to cancer-related cognitive impairment; however, the mechanistic pathways linking metabolic dysfunction, tumor progression, and brain dysfunction remain incompletely defined. Emerging evidence indicates that obesity-induced gut microbial dysbiosis and intestinal barrier disruption may serve as a biologically plausible mechanism connecting these processes via the gut–brain axis although direct clinical causality remains to be firmly established. In obesity, alterations in gut microbiota composition characterized by depletion of barrier-protective taxa and enrichment of pro-inflammatory and genotoxic pathobionts compromise epithelial tight-junction integrity and promote metabolic endotoxemia. The translocation of microbial products, including lipopolysaccharide, sustains chronic systemic inflammation, accelerates CRC progression, and remodels the tumor microenvironment. Notably, these peripheral inflammatory signals extend beyond the intestine and tumor, disrupting blood–brain barrier integrity, activating microglia and astrocytes, and impairing synaptic plasticity within hippocampal and frontal networks. Clinically, these processes manifest as cancer-related cognitive impairment (CRCI), with predominant deficits in attention, processing speed, and working memory, which are often detectable around the time of diagnosis and independent of chemotherapy exposure. This review synthesizes in vivo, in vitro, and human evidence into a proposed theoretical “two-barrier failure” model of obesity-associated CRC and cognitive dysfunction. In addition to mechanistic synthesis, we discuss barrier-centered therapeutic strategies, including targeted probiotics, postbiotics, SCFA supplementation, obesity management through dietary and weight-loss interventions, and potential pharmacological approaches to epithelial and neurovascular barrier protection. We also outline testable clinical trial designs for evaluating these interventions in obesity-associated CRC.

## 1. Introduction

The global incidence of both obesity and colorectal cancer (CRC) has increased markedly over recent decades [1,2]. Obesity is now recognized as a critical environmental risk factor that extends beyond excess weight gain, promoting the development of more than 12 cancer types, including CRC, as well as type 2 diabetes mellitus, nonalcoholic fatty liver disease, and cardiovascular diseases [3,4]. As of 2022, approximately 2.5 billion adults worldwide are estimated to be overweight, with 890 million classified as obese [5,6]. Notably, obesity does not function as a passive background condition; rather, it actively reshapes systemic immunity, endocrine signaling, and microbial ecology through chronic low-grade inflammation (“meta-inflammation”), sustained by immune cell infiltration of adipose tissue and the persistent release of inflammatory mediators [7,8].

This pro-inflammatory milieu not only accelerates tumorigenesis but also adversely affects cognitive function, thereby establishing a mechanistic link among metabolic disease, cancer, and neurodegeneration [9,10]. Of particular clinical relevance is the observation that approximately 45% of patients with CRC exhibit objective cognitive impairment before initiating surgery or chemotherapy [11]. This cancer-related cognitive impairment (CRCI) predominantly manifests as deficits in information processing speed, attention, working memory, and verbal learning capacity, substantially impairing quality of life and social functioning [11,12,13,14]. In CRC, cognitive vulnerability should be considered within a broader cancer-related cognitive impairment framework, in which systemic inflammation, gut-derived immune activation, perioperative stress, and treatment-related factors may collectively shape neurocognitive outcomes, rather than being attributed solely to chemotherapy exposure [15]. To explicitly define the scope of this review, our primary focus is directed toward pathophysiological mechanisms driven by the host and the tumor. These mechanisms specifically include the gut–brain axis and systemic inflammation that occur independently of or prior to the initiation of cancer therapies. While neurotoxicity related to treatment, such as cognitive decline induced by chemotherapy, is a well established and interacting clinical factor, emphasizing vulnerability before treatment allows us to isolate the specific impact of the two barrier failure model, which likely synergizes with subsequent therapeutic interventions. Moreover, longitudinal studies indicate that cognitive deficits may persist for years after treatment completion, with obesity further exacerbating this trajectory through sustained inflammatory signaling [16]. Emerging evidence implicates gut–brain axis dysfunction as a central mechanism underlying obesity-associated cognitive deterioration. In preclinical models, obesity-induced gut microbial dysbiosis has been shown to compromise the integrity of intestinal epithelial tight junctions, which may result in increased intestinal permeability, commonly referred to as intestinal barrier disruption [17,18]. Translocation of bacterial endotoxins, including lipopolysaccharide (LPS), and pro-inflammatory cytokines into the circulation promotes systemic inflammation and can disrupt blood–brain barrier (BBB) integrity or signal via the vagus nerve to induce neuroinflammation, ultimately impairing cognitive function [19,20,21,22,23].

Critically, germ-free animal models demonstrate that the gut microbiota directly regulates BBB integrity: the absence of commensal microbes increases BBB permeability and reduces tight-junction protein expression, phenotypes that are reversible upon microbial colonization [24]. In addition, microbiota-derived short-chain fatty acids (SCFAs), particularly butyrate, support intestinal epithelial metabolism and barrier function by promoting colonocyte energy homeostasis, epithelial hypoxia-inducible factor signaling, and tight-junction integrity [25]. SCFAs also influence BBB integrity and microglial maturation and function in the CNS, thereby serving as key molecular mediators of microbiota–gut–brain communication [26]. Recent studies have also identified specific microbial signatures associated with both CRC and cognitive decline. For example, depletion of *Faecalibacterium prausnitzii* and enrichment of pathobionts such as *Fusobacterium nucleatum* have been reported in obese patients with CRC and are associated with heightened systemic inflammation and poorer neurocognitive outcomes [27,28]. Collectively, these findings suggest that obesity-driven meta-inflammation and gut–brain axis dysfunction may act as contributing factors to CRC-associated cognitive impairment.

Accordingly, this review aims to synthesize experimental, translational, and clinical evidence into a two-barrier failure model of obesity-associated CRC and cognitive dysfunction. We explicitly define intestinal epithelial disruption as the first barrier failure, systemic inflammation as the inflammatory relay, and BBB/neurovascular unit dysfunction as the second barrier failure. Beyond constructing this model, we further examine barrier-centered therapeutic strategies, including microbiome modulation, postbiotic and SCFA-based approaches, obesity management, and pharmacological barrier protection, and we explicitly discuss the limitations and future validation steps required before clinical translation. By organizing the evidence around this sequence, we propose a unifying framework that links obesity-induced dysbiosis and intestinal barrier disruption to CRC progression, neuroinflammation, and cancer-related cognitive impairment. This review aims to (1) propose a testable two-barrier failure model, (2) synthesize stepwise mechanistic and clinical evidence, (3) evaluate therapeutic strategies targeting each model component, and (4) identify testable hypotheses and study designs to validate the model.

## 2. Literature Search Strategy

To ensure a comprehensive and balanced synthesis of the current evidence and mitigate the risk of selection bias, a literature search was conducted using PubMed, Scopus, and Web of Science databases for articles published up to May 2026. The search strategy employed combinations of the following keywords: “obesity,” “colorectal cancer,” “gut microbiota,” “dysbiosis,” “intestinal permeability,” “blood-brain barrier,” “neuroinflammation,” and “cognitive impairment.”

Both preclinical (in vivo and in vitro) and human clinical (observational cohorts, randomized controlled trials) studies were evaluated. We prioritized peer-reviewed articles that provided mechanistic insights or clinical correlations relevant to the gut–brain axis. To explicitly address mechanistic uncertainty, human clinical data were weighted as primary evidence for disease association, whereas preclinical studies were strictly utilized to explore plausible biological pathways where human data remain limited. Furthermore, to provide a balanced perspective, we actively considered and included studies highlighting the limitations, strain-specific variability, or contradictory outcomes of gut-targeted interventions (e.g., probiotics and microbiome modulation).

## 3. Colorectal Cancer

CRC ranks as the third most common malignancy worldwide and represents a leading cause of cancer-related mortality. In 2020, over 1.9 million new cases and 930,000 deaths were reported globally [1]. Obesity is an independent risk factor for CRC, with each 10 kg increase in body weight associated with an approximately 8% increase in CRC risk, demonstrating a clear dose–response relationship [29,30,31]. Notably, this association persists after adjustment for physical activity, dietary factors, and smoking, underscoring obesity as a biologically active driver of colorectal carcinogenesis rather than a mere confounder [7]. Beyond incidence, obesity is associated with adverse CRC outcomes through metabolic stress, immune remodeling, and microbial factors that influence tumor growth and treatment response [32]. Microbial signatures, such as *Fusobacterium nucleatum*, have been associated with recurrence following chemotherapy in human CRC tissues, suggesting that obesity-linked dysbiosis may intersect with treatment resistance pathways [32]. In obese hosts, chronic low-grade inflammation alters myeloid cell polarization and promotes T-cell exhaustion within the tumor microenvironment, fostering immune evasion and aggressive tumor behavior in preclinical models [33]. These findings indicate that microbial enrichment within tumors can directly modulate cancer cell survival pathways and clinical outcomes. The molecular characteristics of CRC are profoundly modulated by the obese microenvironment. CRC is classified into consensus molecular subtypes (CMS1–4) [34,35]. CMS4 tumors are enriched for stromal activation, inflammatory cytokine signaling, and epithelial–mesenchymal transition programs, all of which are potentiated by obesity-driven inflammation [36,37]. In obesity, CRC cells interact with adjacent white adipocytes to induce lipolysis, using the released free fatty acids as an energy source to drive rapid tumor proliferation and metastasis [38,39,40]. In vivo imaging and co-culture experiments demonstrate that adipocyte-derived free fatty acids are transferred to CRC cells, enhancing mitochondrial β-oxidation and promoting tumor growth and invasiveness in both co-culture systems and mouse models [41]. This metabolic coupling establishes a permissive niche for tumor expansion in individuals with obesity. Obesity-associated microbiota can also contribute to CRC mutational processes. Repeated exposure of human intestinal organoids to genotoxic pks^+^ *Escherichia coli* induces a distinct mutational signature that is detectable in subsets of human CRC genomes, supporting a direct microbe-to-mutation route [42]. Complementary in vivo studies show that colonization with pks^+^ *E. coli* increases tumor burden in inflammation-associated CRC mouse models, linking microbial genotoxicity to tumor promotion at the organismal level [43]. Furthermore, obesity induces systemic hormonal imbalances. Elevated circulating levels of insulin and insulin-like growth factor-1 (IGF-1) activate the phosphoinositide 3-kinase (PI3K)/protein kinase B (Akt) signaling pathway, inhibiting cancer cell apoptosis while promoting proliferation [44,45,46]. Experimental inhibition of IGF-1 receptor signaling suppresses colon tumor growth in obese mouse models, implicating this axis in obesity-enhanced CRC progression [47]. Leptin, secreted by adipose tissue, acts as a pro-tumorigenic factor for CRC, whereas adiponectin, which possesses anti-inflammatory and anti-tumor properties, is reduced in patients with obesity, further exacerbating cancer progression [48,49,50,51]. Leptin stimulation activates signal transducer and activator of transcription 3 (STAT3), mitogen-activated protein kinase (MAPK), and nuclear factor kappa B (NF-κB) signaling in colon cancer cells in vitro, promoting proliferation, migration, and resistance to apoptosis, thereby providing mechanistic plausibility for adipokine-driven tumor acceleration under obese conditions [52,53,54]. Conversely, adiponectin suppresses CRC cell growth and inflammatory signaling, highlighting a critical adipokine imbalance that favors tumor aggressiveness in obesity [55,56]. Consistent with this concept, clinical and genetic epidemiologic studies support an association between higher circulating IGF-1 and increased CRC risk, reinforcing IGF-axis activity as a biologically relevant mediator linking obesity and CRC [57].

Collectively, these findings indicate that obesity reshapes CRC biology at multiple levels—including metabolism, immune surveillance, microbial mutagenesis, and hormonal signaling—thereby promoting aggressive tumor phenotypes and poorer clinical outcomes. Notably, many obesity-driven mechanisms generate sustained systemic inflammation, providing a mechanistic foundation for downstream effects on distant organs, including the brain, as discussed in subsequent sections.

## 4. Colorectal Cancer, Intestinal Permeability, and Gut Microbiota in Obesity

**Figure 1:**
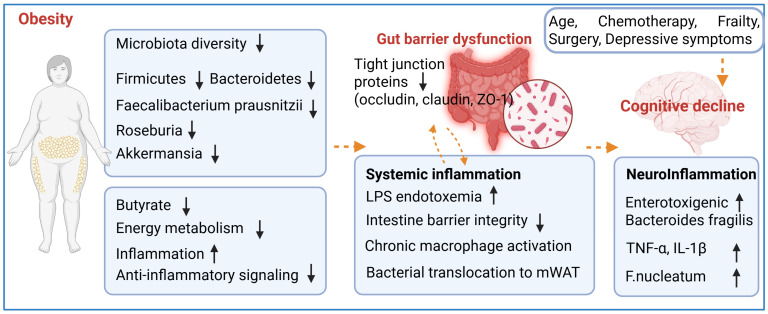
**Proposed Pathways Linking Obesity-associated Gut Microbiota Dysbiosis to Cognitive Decline.** This schematic representation summarizes a multi-step, two-barrier failure model, where dashed arrows indicate proposed mechanisms drawn primarily from preclinical and indirect human evidence rather than strictly established physiological cascades. Initially, obesity-associated dysbiosis, characterized by reduced microbial diversity and an expansion of pathobionts, is hypothesized to contribute to intestinal barrier dysfunction. This increased intestinal permeability potentially enables the translocation of gut-derived inflammatory mediators into the circulation, initiating a systemic inflammatory relay that interfaces bidirectionally with adipose tissue-derived meta-inflammation to further exacerbate gut barrier integrity. Subsequently, this chronic systemic inflammatory burden may promote neurovascular vulnerability, triggering neuroinflammation and synaptic dysfunction that culminate in cognitive decline. Importantly, as highlighted by the clinical confounders box, cancer-related cognitive decline is a highly multifactorial condition heavily influenced by alternative or interacting pathways, including age, chemotherapy, frailty, surgery, and depressive symptoms. Solid arrows (↑/↓) within the panels denote an increase or decrease in specific biological parameters, whereas dashed arrows between the panels represent hypothesized mechanistic links and bidirectional feedback loops that require further prospective clinical validation. **Abbreviations:** ZO-1, zonula occludens-1; LPS, lipopolysaccharide; mWAT, mesenteric white adipose tissue; TNF-α, tumor necrosis factor-alpha; IL-1β, interleukin-1 beta.

Both obesity and CRC are closely associated with gut microbial dysbiosis, which is hypothesized to act as a mediating factor linking metabolic dysfunction, intestinal barrier failure, and tumor-promoting inflammation [2,17]. Diet-induced obesity profoundly alters gut microbial composition, reducing microbial diversity and destabilizing the intestinal ecosystem [58,59]. High-fat diets consistently increase the Firmicutes-to-Bacteroidetes ratio while depleting beneficial taxa, including *Akkermansia muciniphila* and SCFA–producing bacteria, thereby impairing epithelial energy supply and barrier resilience [58,60,61]. SCFAs, particularly butyrate, serve as the primary energy source for colonocytes and are essential for maintaining tight-junction integrity and mucosal immune tolerance; thus, their depletion in obesity directly compromises epithelial homeostasis [62]. Concomitantly, CRC-associated dysbiosis is characterized by enrichment of pathogenic or pro-tumorigenic bacteria, including *Fusobacterium nucleatum* and genotoxic *Escherichia coli*, which accelerate tumor initiation and progression through inflammatory and mutagenic mechanisms. Restoration of *Akkermansia muciniphila* in specific diet-induced obese mouse models improves glucose metabolism, reduces metabolic endotoxemia, and attenuates systemic inflammation, supporting a critical barrier-protective role for this taxon in vivo [63] (Figure 1). More recently, *Akkermansia*-derived extracellular vesicles were shown to directly increase the expression of tight-junction proteins such as zonula occludens-1 (ZO-1) and occludin in intestinal epithelial cells and to restore barrier integrity in high-fat diet models, establishing a clear mechanistic link between microbial activity and epithelial reinforcement [64]. While these mechanistic findings from metabolic models underscore the barrier-protective potential of specific taxa and SCFAs, extrapolating these local intestinal effects to systemic neuroprotection in CRC patients requires caution and distinct clinical validation. In contrast, chronic low-grade inflammation associated with obesity suppresses epithelial tight-junction maintenance. In both in vitro intestinal epithelial cell cultures and murine models, pro-inflammatory cytokines decrease the expression and membrane localization of ZO-1 and occludin, thereby increasing paracellular permeability [20]. Recent mechanistic studies further demonstrate that obesity-associated microbial dysbiosis impairs ethanolamine metabolism, activating microRNA-101a-3p, which directly suppresses ZO-1 expression and exacerbates intestinal permeability in both individuals with obesity and mouse models [21,22]. These findings indicate that intestinal barrier disruption in obesity is not a passive consequence of inflammation but is actively driven by microbiota–epigenetic interactions. CRC-associated pathobionts further intensify epithelial stress and tumor-promoting inflammation. Enterotoxigenic *Bacteroides fragilis* (ETBF) has been shown to induce colonic tumorigenesis specifically in murine models of inflammation-associated CRC [65] (Figure 1). Separately, in human CRC tissues, *Fusobacterium nucleatum* promotes CRC progression and metastasis and is mechanistically associated with chemoresistance. However, it is important to note that while these specific pathobionts strongly drive intestinal and tumor pathology, direct human evidence demonstrating their individual roles in propagating CRC-related cognitive impairment remains currently unavailable [32] (Figure 1). As intestinal barrier integrity deteriorates, Gram-negative bacterial endotoxins, particularly LPS, translocate into the systemic circulation, a process termed “metabolic endotoxemia,” and a hallmark of obesity-associated intestinal barrier disruption [8] (Figure 1). Circulating LPS activates Toll-like receptor 4 (TLR4) signaling in adipose tissue macrophages, promoting M1 polarization and triggering the release of pro-inflammatory cytokines such as interleukin (IL)-1β, IL-6, and tumor necrosis factor-alpha (TNF-α), which further amplify systemic inflammation [66,67] (Figure 1). This cytokine-rich milieu not only accelerates CRC progression but also primes distal organs, including the brain, for inflammatory injury. In obesity, intestinal bacteria can breach the intestinal barrier and directly translocate to adjacent mesenteric white adipose tissue (mWAT), where they establish residence [68,69]. These tissue-resident microbes act as chronic inflammatory foci, sustaining local macrophage activation and serving as persistent sources of systemic inflammatory signals [69]. Notably, viable translocation of gut microbiota to mesenteric adipose tissue has been demonstrated in humans, confirming that this phenomenon is not restricted to rodent models and is likely clinically relevant in obesity-associated inflammatory states [68] (Figure 1).

Collectively, obesity-associated gut microbial dysbiosis and intestinal barrier disruption are proposed to form an interacting cycle where dysbiosis may exacerbate intestinal barrier disruption, systemic inflammation, and CRC-promoting signals. This chronic inflammatory overflow establishes a mechanistic foundation for distant organ dysfunction, including neuroinflammation and cognitive impairment, providing a critical link between obesity, CRC progression, and gut–brain axis pathology, as discussed in subsequent sections.

## 5. The Two-Barrier Failure Model: Conceptual Framework, Mechanistic Sequence, and Testable Predictions

Although the individual components of this axis—obesity, CRC, microbiota disruption, intestinal barrier dysfunction, systemic inflammation, and BBB impairment—are supported by varying degrees of experimental and clinical evidence, the direct continuous sequence linking these events in humans remains to be definitively established. Therefore, we propose the “two-barrier failure” model as a unifying theoretical framework that integrates these distinct observations and generates testable predictions. In this model, cognitive vulnerability in obesity-associated CRC arises from sequential, interdependent, and mutually reinforcing disruption of two anatomically distinct but functionally connected barriers: the intestinal epithelial barrier and the BBB. First, obesity-associated dysbiosis and chronic low-grade inflammation compromise the intestinal barrier, reducing mucus-layer integrity, disrupting tight-junction proteins, and permitting microbial products such as LPS, bacterial DNA, and other pathogen-associated molecular patterns to enter the systemic circulation. Second, these gut-derived signals, together with adipose tissue- and tumor-associated inflammatory mediators, form a systemic inflammatory relay characterized by metabolic endotoxemia and elevated cytokines, including TNF-α, IL-1β, and IL-6. Third, this persistent inflammatory relay is hypothesized to compromise BBB and neurovascular unit integrity, activate endothelial, pericytic, astrocytic, and microglial responses, and promote neuroinflammation. Thus, CRC-related cognitive impairment in patients with obesity may not result from isolated gut dysfunction or isolated brain vulnerability, but rather from cumulative failure of both peripheral and central barrier systems. This model differs from a general gut–brain axis concept by defining a specific barrier-to-barrier pathological sequence. The first barrier failure occurs at the intestinal epithelial interface, where obesity-associated dysbiosis, reduced SCFA production, mucus-layer disruption, and tight-junction impairment facilitate microbial translocation. The second barrier failure occurs at the neurovascular interface, where the resulting systemic inflammatory and metabolic load weakens BBB integrity and increases the susceptibility of neural circuits to inflammatory injury. Between these two barriers lies a systemic inflammatory-metabolic relay composed of microbial products, cytokines, adipose tissue-derived mediators, and CRC-associated tumor inflammatory signals. Thus, the model is not merely a parallel description of gut and brain abnormalities; rather, it proposes a stepwise continuum in which intestinal barrier disruption generates the circulating inflammatory burden that subsequently drives BBB vulnerability and neuroinflammation. The two-barrier failure framework therefore explicitly integrates intestinal barrier failure and BBB dysfunction into a single mechanistic cascade: the intestinal breach increases circulating endotoxin and cytokines while simultaneously reducing protective metabolites such as SCFAs, thereby both increasing the inflammatory load reaching the neurovascular unit and removing metabolite-mediated BBB resilience. This integrated framework generates several falsifiable predictions. First, patients with obesity-associated CRC who exhibit combined evidence of intestinal permeability, systemic endotoxemia, and inflammatory activation should show greater BBB-related biomarker abnormalities and worse cognitive performance than patients with only one altered domain. Second, restoration of SCFA availability or reduction in metabolic endotoxemia should improve intestinal barrier markers and attenuate downstream neuroinflammatory or cognitive outcomes. Third, interventions targeting both the first barrier and the systemic relay are expected to be more effective than strategies directed at a single barrier alone. These predictions provide a rationale for longitudinal biomarker studies and interventional trials incorporating gut permeability, inflammatory, neurovascular, and cognitive endpoints.

### 5.1. First Barrier Failure: Obesity-Induced Intestinal Epithelial Disruption

In the proposed model, intestinal epithelial disruption represents the initiating barrier defect. Obesity-associated dysbiosis reduces barrier-protective metabolites, alters mucus and tight-junction integrity, and increases the likelihood that microbial products enter the circulation, thereby initiating the systemic inflammatory relay. In the first step of the model, obesity acts as a primary driver of intestinal barrier failure. High-fat diet, adipose tissue inflammation, insulin resistance, and altered bile acid metabolism reshape the gut microbiota toward a pro-inflammatory configuration. This dysbiotic state is characterized by reduced abundance of barrier-supportive taxa, impaired production of short-chain fatty acids such as butyrate, thinning of the mucus layer, and reduced expression or altered localization of tight-junction proteins including occludin, claudins, and zonula occludens-1. As epithelial integrity declines, microbial products translocate across the intestinal mucosa, producing metabolic endotoxemia and sustained activation of innate immune pathways. In the context of CRC, this first barrier failure may further amplify tumor-promoting inflammation, epithelial proliferation, immune evasion, and remodeling of the tumor microenvironment.

### 5.2. Systemic Inflammatory-Metabolic Relay

A central component of the two-barrier failure model is the systemic inflammatory relay that links intestinal permeability to BBB dysfunction. Once microbial products cross the impaired intestinal barrier, circulating LPS, peptidoglycans, bacterial DNA, and inflammatory cytokines can activate monocytes, endothelial cells, and hepatic acute-phase responses. In parallel, CRC itself contributes additional inflammatory mediators through the tumor microenvironment, including cytokines, chemokines, prostaglandins, and damage-associated molecular patterns. Obesity further primes this process by maintaining adipose tissue-derived inflammation and oxidative stress. Together, these signals create a chronic inflammatory milieu capable of altering BBB endothelial tight junctions, increasing vascular permeability, and sensitizing resident glial cells to secondary inflammatory stimuli. This relay may operate through circulating endotoxins and cytokines, endothelial activation, hepatic acute-phase responses, complement activation, and leukocyte trafficking, thereby translating intestinal leakage into neurovascular stress.

### 5.3. Second Barrier Failure: BBB and Neurovascular Unit Dysfunction

The second barrier failure occurs at the level of the BBB and neurovascular unit. The BBB is maintained by specialized endothelial cells, tight-junction complexes, pericytes, astrocytic end-feet, basement membrane components, and immune-regulatory signaling within the neurovascular unit. In animal models of endotoxemia, systemic inflammatory mediators generated after intestinal barrier failure can disrupt this structure by downregulating tight-junction proteins, increasing endothelial oxidative stress, activating matrix metalloproteinases, and promoting leukocyte adhesion or transmigration. Increased BBB permeability allows peripheral cytokines and microbial-derived inflammatory signals to influence brain parenchyma more directly, resulting in microglial priming, astrocyte activation, altered synaptic plasticity, and impaired neural network function. These changes provide a plausible mechanistic basis for the attention, processing speed, working memory, and verbal learning deficits observed in CRC-related cognitive impairment.

### 5.4. Neuroinflammatory and Cognitive Consequences

**Figure 2:**
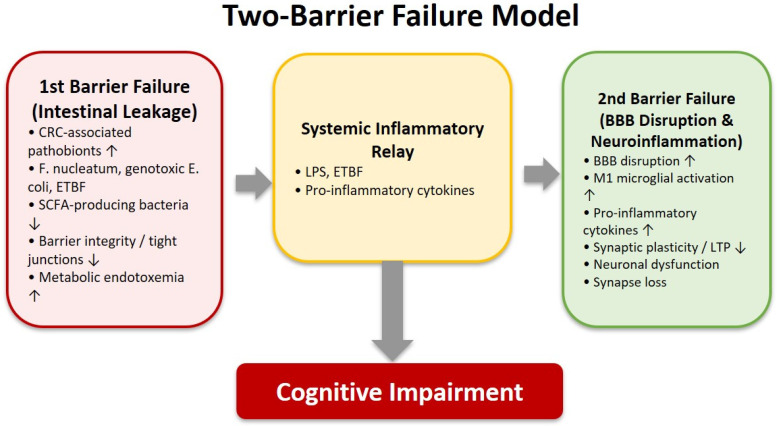
**The two-barrier failure model linking obesity-associated dysbiosis, colorectal cancer progression, and cognitive impairment.** Obesity-induced dysbiosis and CRC-associated pathobionts (e.g., *Fusobacterium nucleatum*, genotoxic *E. coli*, and ETBF) lead to the disruption of tight junctions and a decline in SCFA-producing bacteria, resulting in First Barrier Failure (intestinal leakage). This triggers metabolic endotoxemia and a Systemic Inflammatory Relay via the circulation, transporting LPS, ETBF, and pro-inflammatory cytokines to the brain. Consequently, these mediators provoke Second Barrier Failure (BBB disruption), leading to M1 microglial activation, neuroinflammation, synapse loss, and ultimately, cognitive impairment. Upward arrows indicate an increase, and downward arrows indicate a decrease. **Abbreviations**: ETBF, enterotoxigenic Bacteroides fragilis; SCFA, short-chain fatty acids; LPS, lipopolysaccharide; BBB, blood–brain barrier; LTP, long-term potentiation, ↑ indicate an increase, and ↓ indicate a decrease.

Clinical and experimental evidence supports several components of the two-barrier failure model. In patients with CRC, cognitive impairment can be detected near diagnosis and even before adjuvant therapy, suggesting that cancer-related and host inflammatory factors may contribute to cognitive vulnerability independently of treatment-related neurotoxicity. By delineating these pre-treatment cognitive deficits, this review establishes the baseline structural and inflammatory vulnerabilities driven by the gut–brain axis, ensuring that the proposed model is not purely confounded by the well-documented neurotoxic effects of chemotherapy or surgery. In a large prospective human cohort assessed near diagnosis and before adjuvant therapy, human patients with CRC exhibited a higher incidence of objective cognitive impairment than healthy controls, with deficits predominantly affecting fronto-subcortical networks [23]. Patients with CRC also exhibit specific deficits in verbal memory, information-processing speed, attention, and working memory, consistent with fronto-subcortical dysfunction rather than cortical degeneration typical of conditions such as Alzheimer’s disease [13,70]. These clinical observations support the concept that CRC-related cognitive impairment may reflect inflammation-sensitive network dysfunction rather than a primary neurodegenerative process. Mechanistically, intestinal barrier disruption induced by obesity and CRC may influence brain regions involved in cognition through systemic inflammatory, metabolic, and neuroimmune pathways within the gut–brain axis [11,23]. In diet-induced obesity, gut-derived LPS, a key component of metabolic endotoxemia, elevates systemic inflammatory signaling and acts as a persistent peripheral trigger that modulates CNS immune responsiveness [8]. Chronic peripheral inflammation communicates with the brain through humoral pathways, BBB entry points, and neuroimmune interfaces, thereby predisposing vulnerable networks, such as hippocampal and fronto-striatal circuits, to dysfunction [71]. Experimental models further demonstrate that systemic LPS exposure disrupts BBB integrity through neurovascular-unit mechanisms, with both in vivo and in vitro studies confirming increased BBB permeability following endotoxin challenge [71] (Figure 2). While these preclinical findings are compelling, we hypothesize that LPS may serve as a mechanistic mediator connecting first-barrier failure to second-barrier vulnerability in humans as well. While this review primarily focuses on obesity as an upstream driver, it is important to emphasize that colorectal cancer itself can independently contribute to cognitive impairment. Even in the absence of obesity, the CRC tumor microenvironment actively secretes neuroactive and inflammatory mediators. Notably, tumor-derived circulating factors, including heparan sulfate fragments and tumor-derived extracellular vesicles, can act as damage-associated molecular patterns (DAMPs). These tumor-specific factors can directly activate Toll-like receptors on brain endothelial cells and microglia, providing an independent pathway to BBB disruption and neuroinflammation. Therefore, CRC establishes a baseline oncologic vulnerability in the brain, which is subsequently amplified and exacerbated by obesity-induced intestinal leakage.

However, direct translational evidence in CRC cohorts is required to fully validate this link.

At the cellular level, LPS and pro-inflammatory cytokines derived from the gut can compromise BBB integrity and activate microglia, the brain’s resident immune cells [23,72]. LPS-activated microglia may exacerbate BBB dysfunction by increasing oxidative stress signaling via NADPH oxidase, thereby providing a cellular mechanism linking endotoxemia to neurovascular barrier failure [73]. Astrocyte-mediated inflammatory signaling provides an additional mechanism linking systemic inflammation to cognitive dysfunction. TNF-α, in particular, activates astrocytic receptors and directly impairs hippocampal synaptic plasticity and memory [72]. Mechanistic studies demonstrate that TNF-α elevation in the hippocampal dentate gyrus activates astrocytic TNFR1 signaling, triggers an astrocyte–neuron cascade, persistently alters excitatory synapses, and impairs learning and memory [74]. Together, these findings support a pathway in which peripheral inflammation, amplified by intestinal barrier disruption, promotes CNS cytokine signaling, glial activation, and synaptic dysfunction relevant to cognition. In addition to inflammatory mediators, microbial metabolites may regulate BBB resilience. SCFAs, such as butyrate, which are often depleted in obesity, typically maintain BBB integrity and provide neuroprotection; therefore, their deficiency may increase cerebral vulnerability to inflammatory insults. Germ-free mice exhibit increased BBB permeability and reduced tight-junction protein expression, whereas microbial colonization restores BBB integrity, demonstrating a causal gut-to-BBB regulatory axis [24]. Translational studies further show that SCFA supplementation improves BBB function in animal models of dysbiosis, reinforcing the concept that loss of microbial metabolites diminishes BBB resilience [75]. Therefore, obesity-associated intestinal barrier disruption may amplify neuroinflammation through a dual mechanism: increased systemic endotoxin/cytokine burden and reduced metabolite-mediated BBB protection [24] (Figure 2 and Table 1). Neuroimaging and clinical observations further support the network-level consequences of this inflammatory cascade. Neuroimaging studies indicate network-level abnormalities in CRC-related cognitive decline, aligning with inflammation-driven functional dysconnectivity rather than focal cortical pathology [12]. This maladaptive mechanism may disrupt regional network synchronization and correlate negatively with verbal memory performance, without implying equivalence to primary neurodegenerative disease [12,76,77]. In some cases, patients present with acute behavioral changes and acute cognitive decline months before CRC diagnosis, likely reflecting cancer-derived inflammatory mediators acting through the gut–brain axis on the hippocampus and limbic system, with marked improvement observed following cancer treatment [78,79]. Given that tumor-associated microbes, such as *Fusobacterium nucleatum*, influence inflammatory tone and therapeutic response in CRC, future clinical studies should assess whether microbial burden correlates with cognitive trajectories during diagnosis and treatment. It is critical to acknowledge that the proposed two-barrier failure model does not operate in isolation. Clinically, CRC-related cognitive impairment is a highly multifactorial phenomenon. Cognitive trajectories in CRC patients are profoundly influenced by competing and interacting factors, including advanced age, baseline frailty, cancer burden, comorbid vascular diseases, and systemic manifestations such as anemia and sarcopenia. Furthermore, the psychosocial burden of cancer (e.g., depression, sleep disturbances) and treatment-related toxicities from surgery, general anesthesia, chemotherapy, and concurrent medications continuously overlap with the gut–brain axis. Therefore, intestinal barrier disruption and subsequent neuroinflammation should not be viewed as the sole unified explanation for CRCI, but rather as a significant and interacting mechanistic contributor within a broader, complex clinical landscape.

Importantly, the microbiota components discussed herein—such as *F. nucleatum*, *pks+ E. coli*, ETBF, *Akkermansia*, SCFAs, and LPS—should not be interpreted as forming a single, continuous, and universally applicable pathway across obesity, CRC, and cognition. Current evidence is drawn from highly divergent models, populations, and experimental endpoints. For instance, while LPS endotoxemia represents a generalized systemic inflammatory trigger, the oncogenic specificities of *pks+ E. coli* or chemoresistance driven by *F. nucleatum* have distinct, localized roles within the tumor microenvironment. Therefore, we emphasize that these organisms and metabolites act through parallel, intersecting, and sometimes independent mechanisms, rather than a singular unified chain of events, unless explicitly supported by direct human multi-omics data.

### 5.5. Feed-Forward Amplification and Testable Predictions

Taken together, the proposed framework suggests a feed-forward interaction among intestinal barrier dysfunction, systemic inflammatory signaling, BBB vulnerability, and downstream neurocognitive impairment. Rather than implying a fixed linear sequence in all patients, this model is intended to generate testable predictions regarding biomarker coupling across the gut, circulation, and brain. Specifically, it predicts that intestinal permeability markers, systemic inflammatory signatures, microbiome profiles, and BBB-related indicators may converge with longitudinal cognitive trajectories in biologically defined CRC subgroups. Integrative biomarker studies of this nature may identify high-risk subgroups and inform combined interventions targeting barrier repair, microbial metabolites, systemic inflammation, and neurovascular protection to preserve cognition during CRC management. Accordingly, the two-barrier failure model provides the conceptual backbone of this review: intestinal barrier failure explains how obesity-associated dysbiosis becomes systemic, whereas BBB failure explains how systemic inflammation becomes neurocognitive vulnerability in patients with CRC.

## 6. Therapeutic Implications Targeting the Gut–Brain Axis

Here, we expand therapeutic discussion into four mechanistic categories aligned with the two-barrier failure model: (1) microbiome modulation (probiotics, prebiotics, fecal microbiota transplantation and engineered microbes); (2) postbiotic and SCFA-based strategies (butyrate, tributyrin, propionate formulations and butyrate prodrugs); (3) obesity management and metabolic modulation (dietary fiber, bariatric/metabolic surgery, GLP-1 receptor agonists) that reduce adipose-derived inflammation and endotoxemia; and (4) pharmacological or device-based approaches to directly strengthen epithelial and neurovascular barriers (tight-junction stabilizers, anti-TNF/TNF-modulating strategies, repurposed barrier protectors). For each category we summarize mechanistic rationale, preclinical evidence, current clinical data, safety considerations in oncology populations, and proposed next-step trials.

Accordingly, the interventions discussed below should be viewed primarily as mechanism-linked and hypothesis-generating research directions rather than evidence-based clinical recommendations for preserving cognition in patients with CRC. Where human data are available, they largely concern metabolic, inflammatory, microbial, or barrier-related endpoints rather than direct neurocognitive outcomes. From a translational perspective, these approaches can be grouped into three broad levels: upstream reduction in obesogenic and inflammatory burden, microbiome- and metabolite-directed support of barrier integrity, and protection of neurovascular vulnerability. Operating under the hypothesis that intestinal barrier disruption may represent an upstream event converting microbial dysbiosis into systemic and central nervous system pathology, interventions are best conceptualized along a continuum: (i) reducing the obesogenic and inflammatory burden that initiates barrier failure, (ii) restoring a barrier-protective microbial ecosystem, (iii) supplying the microbial metabolites that directly reinforce both barriers, and (iv) translating these strategies into oncology-specific supportive care. The following subsections summarize current experimental and clinical evidence for each of these complementary approaches, while emphasizing that most data remain mechanistic or derived from non-oncologic populations and therefore require validation in patients with obesity-associated colorectal cancer (CRC). In addition to caloric restriction, structured physical activity and exercise constitute a critical component of obesity management that independently modulates the gut–brain axis. Exercise has been shown in murine models to enrich butyrate-producing taxa, enhance intestinal tight-junction expression, and reduce baseline endotoxemia [80,81]. Furthermore, exercise-induced myokines such as irisin can cross the BBB, promoting neurogenesis and directly countering neuroinflammation [82]. Thus, integrating targeted exercise regimens may synergistically protect both the first and second barriers. Accordingly, the interventions discussed below should be viewed primarily as mechanism-linked and hypothesis-generating research directions rather than evidence-based clinical recommendations for preserving cognition in patients with CRC. Where human data are available, they largely concern metabolic, inflammatory, or barrier-related endpoints rather than direct neurocognitive outcomes.

### 6.1. Obesity Management as an Upstream Intervention

Because excess adiposity is a proximal driver of meta-inflammation and intestinal barrier disruption, weight reduction can be considered an upstream risk-modifying strategy within this framework. In a controlled study of Roux-en-Y gastric bypass, sustained weight loss was accompanied by lower circulating LPS and pro-inflammatory cytokine concentrations, together with increased colonic expression of the tight-junction proteins ZO-1 and claudin-1; notably, intestinal permeability correlated positively with LPS levels and inversely with tight-junction protein expression, mechanistically linking weight loss to barrier repair and reduced endotoxemia [83]. These findings support the concept that obesity management does not merely improve metabolic indices but actively reverses the structural and immunological substrate of the first barrier failure. From a neurocognitive standpoint, lower-grade metabolic endotoxemia would be expected to relieve the chronic inflammatory pressure on the BBB, although direct evidence that surgical or dietary weight loss improves cognition in patients with CRC is currently lacking and constitutes an important research gap. Because obesity acts upstream of gut dysbiosis and systemic inflammation, obesity management can be considered a biologically plausible upstream intervention within this framework. Weight reduction, dietary intervention, physical activity, and improved insulin sensitivity may reduce adipose-derived inflammatory signaling, restore microbial diversity, and decrease metabolic endotoxemia, thereby lessening upstream barrier stress. However, direct evidence that obesity-targeted interventions preserve cognition specifically in CRC populations remains limited, and this concept should currently be regarded as an upstream translational hypothesis rather than a validated therapeutic strategy.

### 6.2. Microbiome Modulation: Probiotics and Barrier-Protective Taxa

Restoring barrier-protective commensals—particularly *Akkermansia muciniphila* and short-chain fatty acid (SCFA)–producing bacteria depleted in obesity—is a biologically plausible and increasingly investigated strategy. In a proof-of-concept human study, daily supplementation with live or pasteurized *A. muciniphila* in volunteers with overweight or obesity was safe and improved insulin sensitivity, plasma lipids, and markers of metabolic endotoxemia [84]. This signal has since been refined: a randomized controlled trial demonstrated that pasteurized *A. muciniphila* supplementation supported weight-loss maintenance in adults with overweight or obesity following a low-energy diet [85], and a more recent clinical trial reported that the metabolic efficacy of *A. muciniphila* supplementation in patients with overweight/obese type 2 diabetes depends on its baseline gut abundance—identifying a microbial-stratified responder phenotype that may help inform future precision-based intervention studies [86]. Mechanistically, these clinical observations are consistent with experimental work showing that *Akkermansia*-derived extracellular vesicles upregulate tight-junction proteins such as ZO-1 and occludin, thereby reinforcing epithelial barrier integrity. Complementary prebiotic approaches that selectively enrich *A. muciniphila* have also improved gut-health indices in randomized controlled settings [87], underscoring that the barrier-protective microbial niche can be modulated through both direct administration and substrate-based enrichment. Probiotics and microbiome-directed interventions may target the first barrier failure by restoring epithelial integrity, enhancing mucus-layer function, increasing SCFA-producing bacteria, and reducing the abundance of pathobionts associated with CRC progression. However, their effects are likely strain-specific and host-context dependent, and careful consideration is required in patients undergoing surgery, chemotherapy, or immunotherapy. More intensive microbiome-directed approaches, including fecal microbiota transplantation and engineered microbes, remain highly investigational in obesity and oncology. Likewise, pharmacological regulation of intestinal permeability remains a theoretical strategy in this context. At present, direct evidence that these approaches improve cognition in CRC populations is lacking [88,89]. Repurposing or developing similar pharmacological barrier protectors could theoretically limit the initial translocation of endotoxins, thereby halting the systemic inflammatory relay before it reaches the BBB. However, direct evidence that these approaches improve cognition in CRC populations remains lacking. Despite encouraging mechanistic and early clinical signals, direct evidence for probiotic-mediated cognitive benefit in CRC patients is currently lacking.

### 6.3. Postbiotics and Short-Chain Fatty Acid Supplementation: Bridging the Two Barriers

Among candidate interventions, SCFAs—and butyrate in particular—are of particular mechanistic interest because they may influence both the intestinal barrier and the BBB, thereby providing a plausible molecular bridge across the gut–brain axis. At the intestinal interface, butyrate is the principal energy source for colonocytes and promotes tight-junction assembly, whereas at the neurovascular interface SCFAs maintain BBB integrity and exert anti-inflammatory effects on microglia [75]. Preclinical studies in rodent models provide supportive mechanistic evidence: prebiotic-driven elevation of SCFAs and their receptors repaired both the intestinal barrier and the BBB and alleviated behavioral deficits in a fecal-microbiota-transplantation model [90], while these animal studies highlight the biological plausibility of targeting the gut–brain axis, further clinical trials are necessary to determine whether SCFA supplementation can replicate these barrier-protective effects in patients with obesity-associated CRC [91]. Mechanistically, butyrate has been shown to regulate BBB transport and intra-endothelial handling of pathogenic peptides in models of neurodegeneration, with butyrate supplementation improving cognition and lowering pathological burden in animal studies [92]. Related metabolic substrates reinforce the same axis: β-hydroxybutyrate facilitates BBB repair through epigenetic upregulation of ZO-1 in experimental stroke [93] and restores barrier function in human iPSC-derived brain microvascular endothelial cells [94]. Collectively, these data suggest that postbiotic and SCFA-based interventions are of particular mechanistic interest because they may influence both intestinal and neurovascular barrier biology. SCFAs, particularly butyrate, provide a mechanistic bridge between intestinal and BBB protection. Butyrate supports intestinal epithelial energy metabolism, promotes tight-junction assembly, and exerts anti-inflammatory effects through histone deacetylase inhibition and regulatory T-cell induction. At the neurovascular level, SCFAs can strengthen BBB integrity, modulate microglial maturation, and reduce inflammatory signaling, suggesting that metabolite-based strategies may influence both intestinal and neurovascular barrier function.

### 6.4. Dietary Modulation and Oncology-Specific Translation

Dietary strategies that reduce obesogenic exposure and increase fermentable fiber may provide a practical means of supporting SCFA-producing taxa and barrier resilience, complementing the microbiome- and metabolite-directed approaches discussed above. Importantly, microbiome modulation is also being explored within oncology itself: a randomized controlled trial of postbiotics in patients with cancer demonstrated favorable shifts in the gut microbiota and metabolome—including enrichment of *A. muciniphila* and *Bifidobacterium* alongside suppression of pathogenic *Escherichia–Shigella*—and an associated reduction in cancer-related pain [95]. While the primary endpoint in that trial was symptomatic rather than cognitive, it provides proof-of-principle that dysbiosis-related pathways may be modifiable in patients with cancer. Translating these observations to cognition will require interventional trials in obesity-associated CRC that incorporate barrier-permeability biomarkers (e.g., LPS and LPS-binding protein), stool and tumor microbiome profiling, and validated neurocognitive endpoints, so that barrier-repair strategies—probiotics, postbiotics, SCFA supplementation, and structured weight management—can be evaluated for their capacity to preserve cognition alongside conventional oncologic outcomes. Despite mechanistic plausibility demonstrated in animal models, unproven clinical causality in CRC remains a key limitation. Future trials must integrate stool and tumor microbiome profiling, intestinal permeability markers, systemic cytokines, BBB-related biomarkers, and longitudinal neurocognitive assessments to determine whether barrier-targeted interventions can indeed preserve cognition during CRC management.

### 6.5. Summary and Therapeutic Outlook

Taken together, the available evidence is consistent with a staged, mechanism-based theoretical framework involving upstream reduction in endotoxemia, microbiome modulation, and metabolite-based support of barrier function. However, the strongest causal data remain preclinical or are derived from metabolic rather than oncologic cohorts. Accordingly, these strategies should currently be regarded as biologically plausible and hypothesis-generating, serving primarily to guide biomarker-driven and interventional research rather than routine clinical implementation.

## 7. Limitations and Future Directions

To critically appraise the current literature, it is essential to recognize the varying levels of evidence and distinct limitations supporting each link within this proposed framework. While the link between obesity and gut permeability is supported by robust in vivo and human observational data, the precise causal epigenetic and microbial mechanisms in humans remain only partially understood. Similarly, the pathway from gut permeability to colorectal cancer progression is primarily supported by murine models of inflammation-associated CRC and in vitro organoid studies, currently lacking direct longitudinal validation in humans. Consequently, although each component is supported by experimental or clinical evidence, direct longitudinal data demonstrating the complete sequence—from obesity-induced intestinal leakage to BBB disruption and cognitive decline—in patients with CRC remains limited. Most available studies examine individual components rather than the entire barrier-to-barrier continuum.

Furthermore, evaluating the association between CRC and cognitive impairment presents significant clinical challenges. While cognitive decline is well-documented in human cross-sectional and prospective cohort studies, it is difficult to disentangle the specific effects of gut–brain axis dysfunction from a multitude of competing clinical factors. Cognitive impairment in CRC patients is deeply intertwined with age, frailty, cancer burden, comorbid vascular disease, surgery, medications, treatment-related neurotoxicity, fatigue, anemia, sleep disturbance, depression, sarcopenia, and nutritional deficiency. Methodological constraints also limit current interpretations. At the neurovascular interface, the capacity of LPS and inflammatory cytokines to induce BBB dysfunction is strongly supported by animal models of endotoxemia; however, BBB dysfunction in humans is difficult to assess directly. Available clinical measures, including neuroimaging indices, circulating endothelial or inflammatory markers, and CSF-based assessments, provide only indirect estimates of neurovascular integrity and are not routinely incorporated into CRC cohorts. Additionally, the mechanistic causality linking specific microbiota to cognitive changes is largely derived from fecal microbiota transplantation studies in rodents, with corresponding human evidence currently restricted to associative correlations. This is further complicated by the fact that microbiome composition in patients with CRC is highly heterogeneous and influenced by diet, antibiotics, bowel preparation, surgery, chemotherapy, immunotherapy, tumor location, geography, and host metabolic status, heavily limiting the generalizability of microbiome-based conclusions.

Finally, although probiotics, postbiotics, SCFAs, and obesity management represent biologically plausible strategies for restoring barrier function, clinical trials evaluating their specific effects on cognitive outcomes in CRC patients are scarce. Their efficacy, optimal timing, safety, and interactions with oncologic therapies require careful evaluation. Future prospective studies should therefore integrate stool and tumor microbiome profiling, intestinal permeability markers, systemic cytokine signatures, BBB-related biomarkers, neuroimaging, and longitudinal neurocognitive assessments to rigorously test the proposed model and identify patients most likely to benefit from barrier-targeted interventions.

Limitations of the two-barrier failure model While the model integrates multiple strands of preclinical and translational evidence, several key limitations must be acknowledged: (1) Most direct causal evidence is from rodent or in vitro models and may not fully generalize to humans with CRC; (2) temporal sequence in humans (intestinal leakage → systemic inflammation → BBB disruption → cognitive decline) has not been demonstrated prospectively; (3) tumor- and treatment-related confounders (surgery, chemotherapy, immunotherapy) may independently influence systemic inflammation and cognition; (4) heterogeneity in microbiome composition and host genetics implies that only specific patient subgroups may follow the proposed sequence. We therefore present the model as hypothesis-generating and propose specific longitudinal and interventional studies to address these gaps.

## 8. Conclusions

This review proposes a two-barrier failure framework to integrate evidence linking obesity-associated dysbiosis, intestinal barrier dysfunction, systemic inflammation, and neurocognitive vulnerability in CRC. Importantly, this framework should be interpreted as hypothesis-generating rather than as an established physiological mechanism, because the direct sequential pathway in humans remains incompletely defined. Accordingly, barrier-centered approaches targeting intestinal dysfunction and microbial dysbiosis should be viewed as plausible translational research directions rather than current clinical recommendations. Future biomarker-linked longitudinal and interventional studies are needed to determine whether such strategies can meaningfully preserve cognition during CRC management.

## Figures and Tables

**Table 1:** Stepwise Components of the Two-Barrier Failure Model Linking Obesity-Associated Gut Dysbiosis, Colorectal Cancer, and Cognitive Impairment.

Model Step	Barrier/Compartment	Key Mechanism	Principal Mediators	Pathological Consequence	Relevant References
Step 1. Obesity-driven dysbiosis	Gut microbiota	Reduced microbial diversity; loss of SCFA-producing and barrier-protective taxa; expansion of pro-inflammatory/pathobiont bacteria	Reduced butyrate and other SCFAs; altered bile acids; increased LPS-producing bacteria	Priming of intestinal inflammation and epithelial vulnerability	Preclinical: [32,42,43,58,60,62,65] Human: [27,28,32,59,61]
Step 2. First barrier failure	Intestinal epithelial barrier	Tight-junction disruption, mucus layer impairment, increased epithelial permeability	ZO-1, occludin, claudins, mucins, LPS	Gut leakage and microbial translocation	Preclinical: [17,18,24,58,60,62,65] Human: [17,18,59,61]
Step 3. Systemic inflammatory relay	Circulation, adipose tissue, immune system, liver	Activation of innate immunity and acute-phase responses; interaction with adipose tissue-derived inflammation	LPS, LBP, TLR4/NF-κB, TNF-α, IL-1β, IL-6, CRP	Metabolic endotoxemia and chronic systemic inflammation	Preclinical: [19,21,22,23,72,73] Human: [20,27,28]
Step 4. CRC amplification loop	Colorectal tumor microenvironment	Tumor-promoting inflammation, immune remodeling, microbial enrichment, and therapy resistance	*Fusobacterium nucleatum*, enterotoxigenic Bacteroides fragilis, cytokines, prostaglandins	CRC progression and amplification of systemic inflammatory burden	Preclinical: [42,43,58,60,62,65] Human: [59,61]
Step 5. Second barrier failure	BBB and neurovascular unit	Endothelial activation, tight-junction loss, oxidative stress, pericyte/astrocyte dysfunction	TNF-α, IL-1β, IL-6, ROS, MMPs, reduced SCFAs	Increased BBB permeability and neurovascular vulnerability	Preclinical: [23,24,72,73,74,75]
Step 6. CNS inflammatory response	Brain parenchyma, microglia, astrocytes, synapses	Microglial priming, astrocyte activation, altered synaptic plasticity, network dysconnectivity	NF-κB, cytokines, oxidative stress, complement signaling	Attention, processing speed, working memory, and verbal learning deficits	Preclinical: [72,73,74,75] Human: [11,12,76,77,78,79]
Step 7. Therapeutic implications	Gut–brain barrier axis	Barrier restoration and inflammatory load reduction	Weight loss, diet, probiotics, prebiotics, postbiotics, SCFAs, anti-inflammatory strategies	Potential preservation of oncologic and cognitive outcomes	Preclinical: [80] Human: [81,82,83,84]

## Data Availability

Data sharing is not applicable to this article as no new data were created or analyzed in this study.

## Abbreviation

Colorectal cancer (CRC); cancer-related cognitive impairment (CRCI); lipopolysaccharide (LPS); blood–brain barrier (BBB); short-chain fatty acids (SCFAs); consensus molecular subtypes (CMS1–4); insulin-like growth factor-1 (IGF-1); phosphoinositide 3-kinase (PI3K)/protein kinase B (Akt); signal transducer and activator of transcription 3 (STAT3); mitogen-activated protein kinase (MAPK); zonula occludens-1 (ZO-1); Enterotoxigenic *Bacteroides fragilis* (ETBF); interleukin-17 (IL-17); Toll-like receptor 4 (TLR4); Interleukin (IL)-1β; tumor necrosis factor-alpha (TNF-α); mesenteric white adipose tissue (mWAT).
